# Supplementary material for: The impact of war on HIV/AIDS service provision: In rural health facilities of Tigray, northern Ethiopia, a cross-sectional study
Source: PLoS One. 2023 May 2;18(5):e0278976. doi: 10.1371/journal.pone.0278976 (PMC10153695; doi:10.1371/journal.pone.0278976)
Supplement: Attachment — Submitted filename: MHZ review on impact of war on HIV .docx [file pone.0278976.s002.docx]

Abstract:

Line 31. is HIV problem for all the world? Make some specific phrasings like in developing country.

Line 51. Key word ART and treatment are same (better to delate duplication)

Line 54. Same comment to line 31

Line 56. Your reference for ‘The impact of HIV is highly pronounced in war-affected settings, including Tigray, northern Ethiopia.’

Methodology

Line 104-105 why purposive selection of 25 out of 84 how to be sure that these 25 represent the rest 84?

Again, in line 106-107, how random selection of 33 can give the image of the whole health center unless systematic sampling made?

Line 111. Do the selected health facilities have complete information for inclusion? If not how much…

Line 129 I don’t think the operational definition is important.

Result and discussion

All the data was analyzed in so simple calculator no Stastically analysed which make it not a research article so, I suggest better to be short report rather than full research article.
