# Supplementary material for: The impact of war on HIV/AIDS service provision: In rural health facilities of Tigray, northern Ethiopia, a cross-sectional study
Source: PLoS One. 2023 May 2;18(5):e0278976. doi: 10.1371/journal.pone.0278976 (PMC10153695; doi:10.1371/journal.pone.0278976)
Supplement: Attachment — Submitted filename: Dear editor.docx [file pone.0278976.s004.docx]

Dear editor,

It is regarding the manuscript ‘The Impact of War on HIV/AIDS Service Provision: in Rural Health Facilities of Tigray, northern Ethiopia, a Cross-sectional Study’.

I can say all my first comments and questions were amended correctly. So my saying is to accept for publication.

Best
